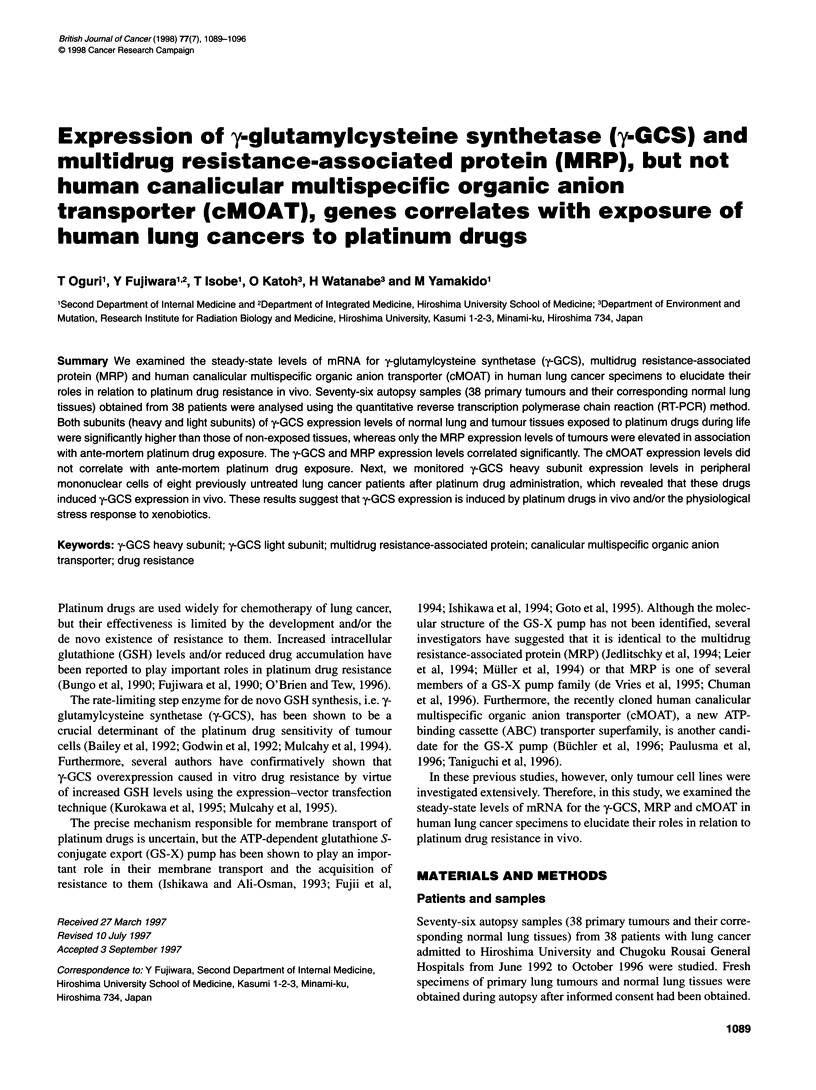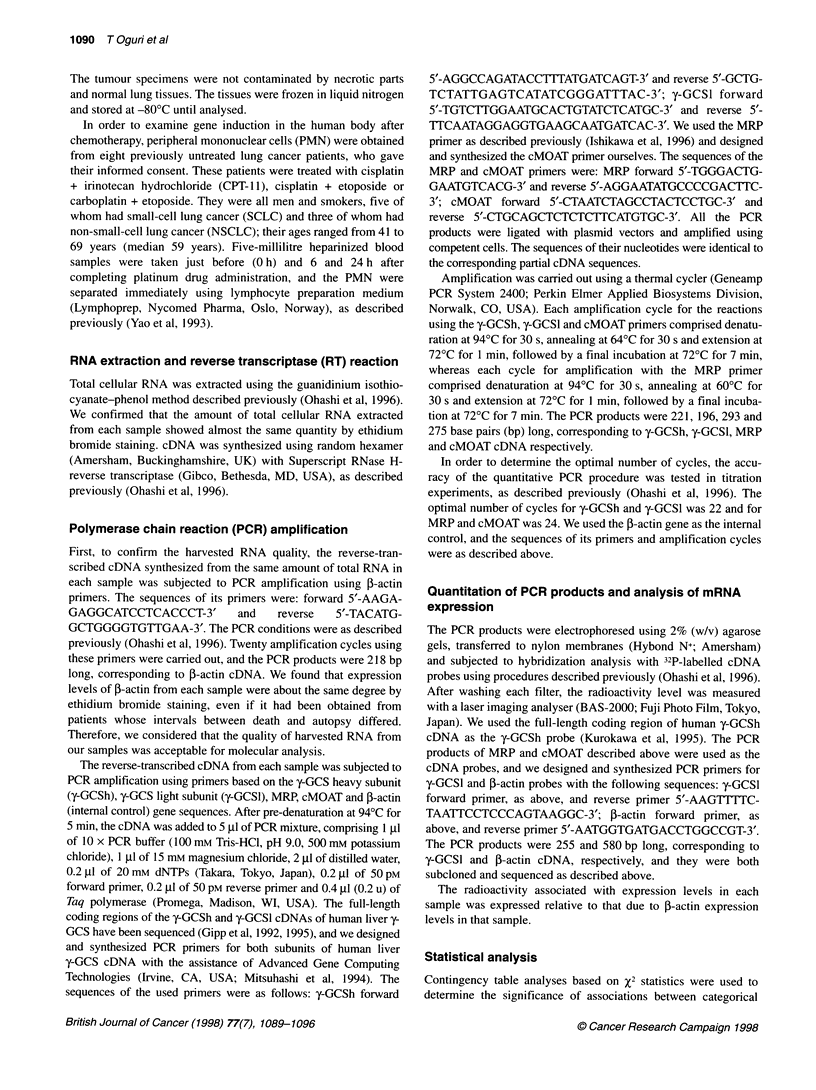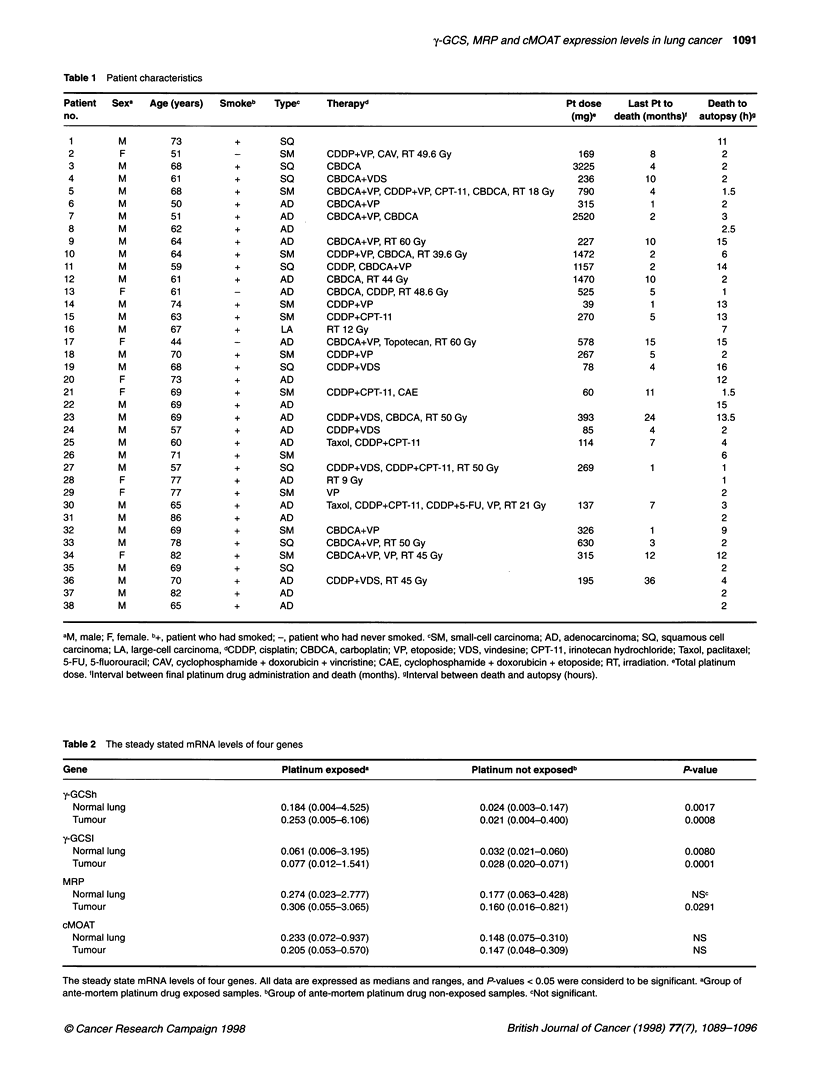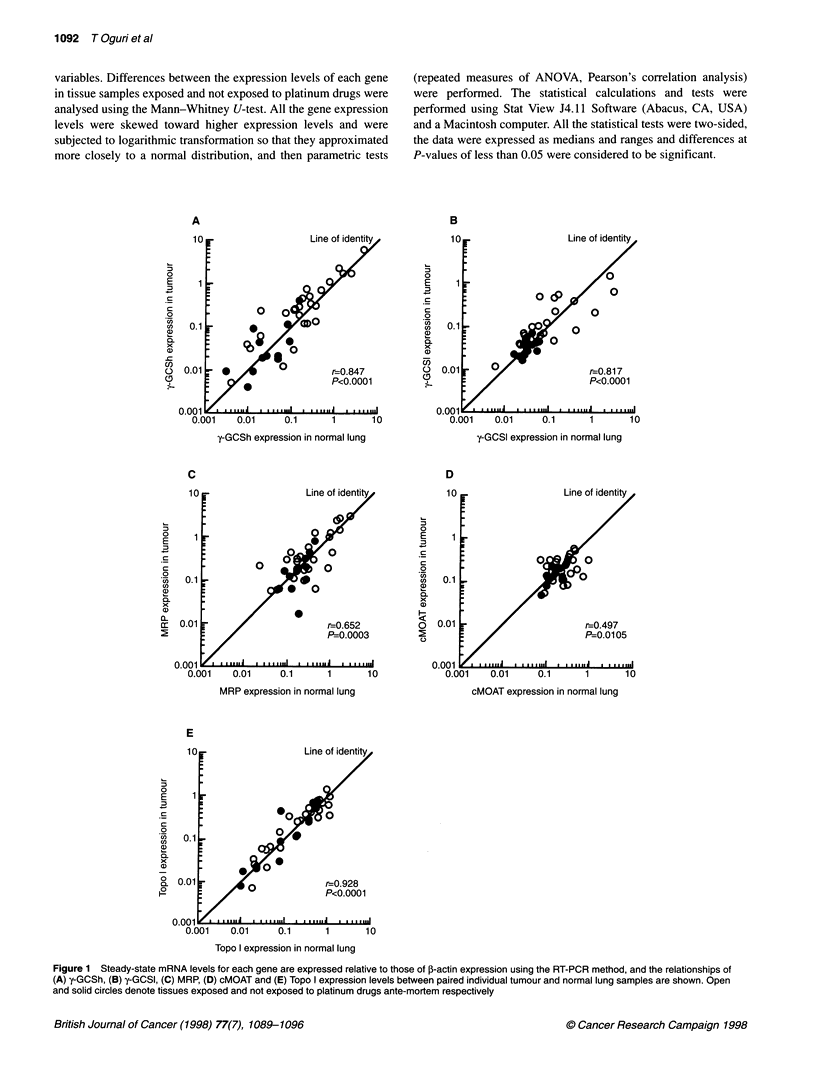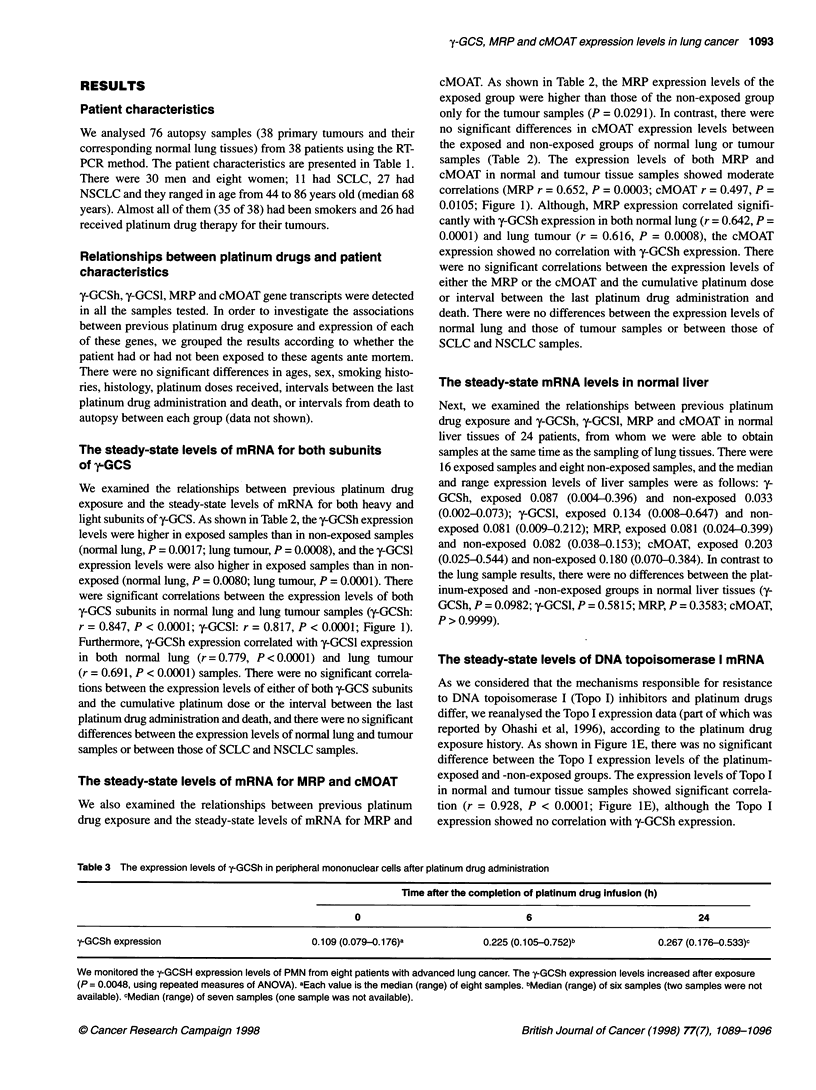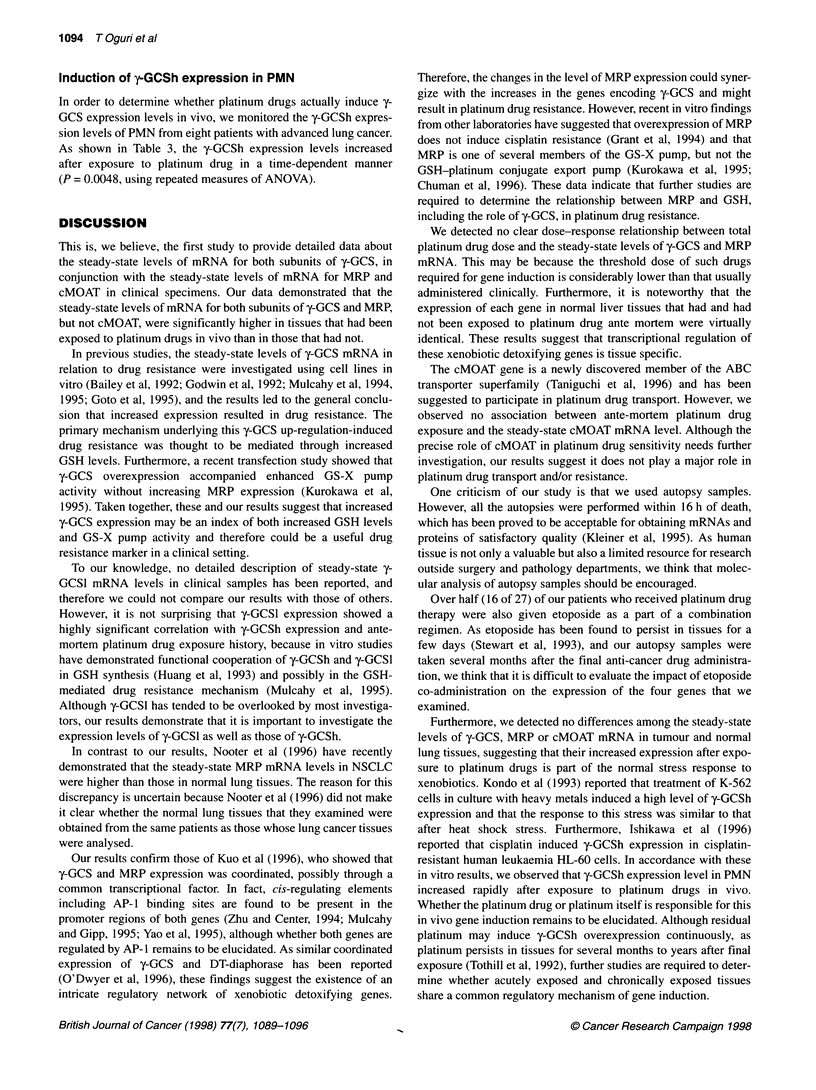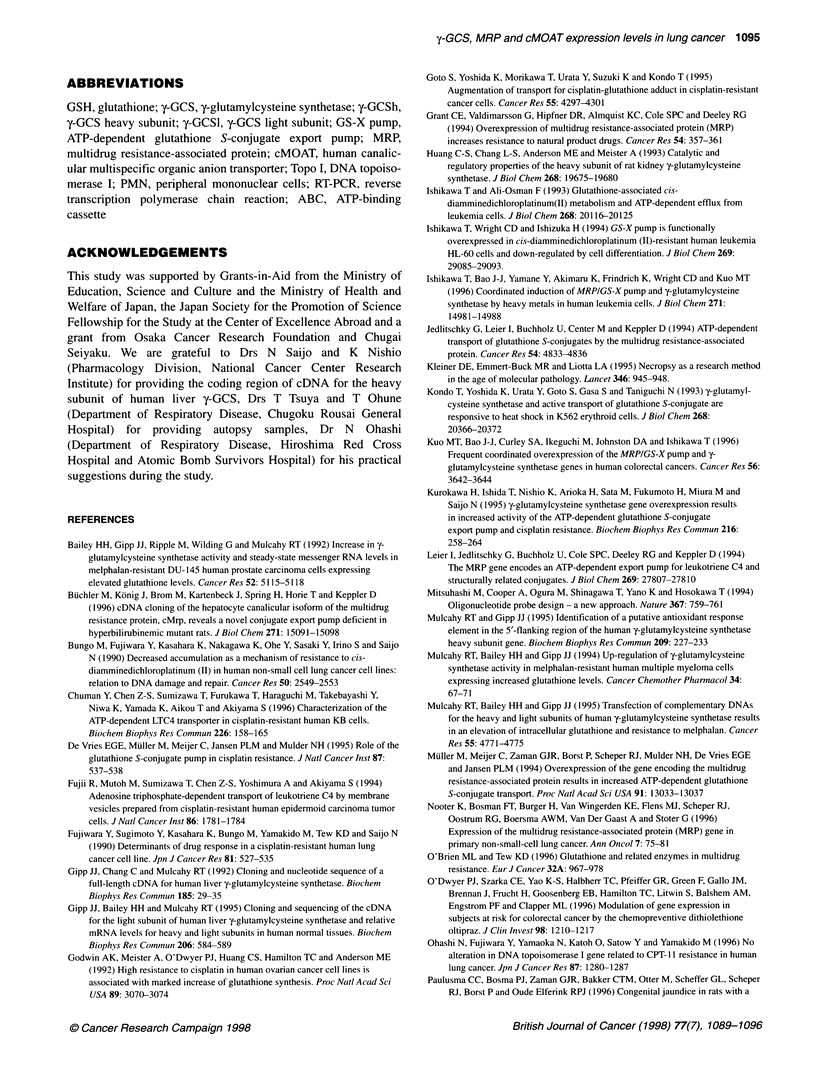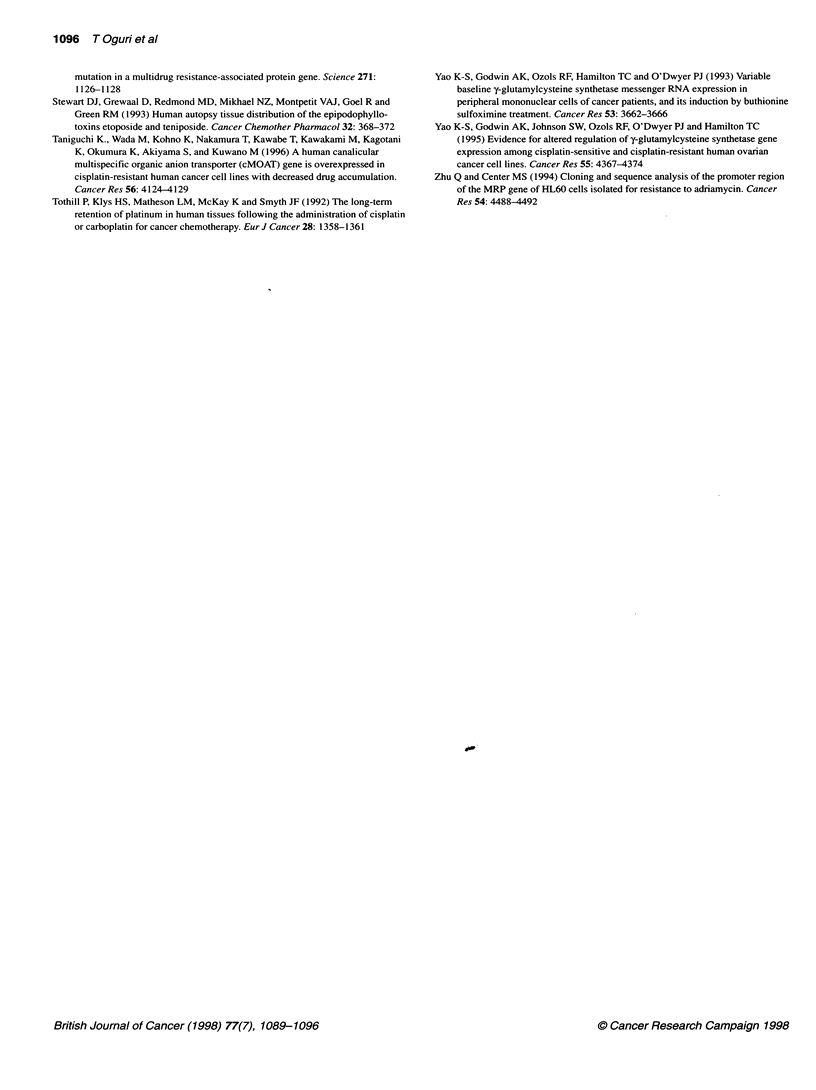# Expression of gamma-glutamylcysteine synthetase (gamma-GCS) and multidrug resistance-associated protein (MRP), but not human canalicular multispecific organic anion transporter (cMOAT), genes correlates with exposure of human lung cancers to platinum drugs.

**DOI:** 10.1038/bjc.1998.181

**Published:** 1998-04

**Authors:** T. Oguri, Y. Fujiwara, T. Isobe, O. Katoh, H. Watanabe, M. Yamakido

**Affiliations:** Second Department of Internal Medicine, Hiroshima University School of Medicine, Japan.

## Abstract

We examined the steady-state levels of mRNA for gamma-glutamylcysteine synthetase (gamma-GCS), multidrug resistance-associated protein (MRP) and human canalicular multispecific organic anion transporter (cMOAT) in human lung cancer specimens to elucidate their roles in relation to platinum drug resistance in vivo. Seventy-six autopsy samples (38 primary tumours and their corresponding normal lung tissues) obtained from 38 patients were analysed using the quantitative reverse transcription polymerase chain reaction (RT-PCR) method. Both subunits (heavy and light subunits) of gamma-GCS expression levels of normal lung and tumour tissues exposed to platinum drugs during life were significantly higher than those of non-exposed tissues, whereas only the MRP expression levels of tumours were elevated in association with ante-mortem platinum drug exposure. The gamma-GCS and MRP expression levels correlated significantly. The cMOAT expression levels did not correlate with ante-mortem platinum drug exposure. Next, we monitored gamma-GCS heavy subunit expression levels in peripheral mononuclear cells of eight previously untreated lung cancer patients after platinum drug administration, which revealed that these drugs induced gamma-GCS expression in vivo. These results suggest that gamma-GCS expression is induced by platinum drugs in vivo and/or the physiological stress response to xenobiotics.


British Journal of Cancer (1998) 77(7), 1089–1096
© 1998 Cancer Research Campaign

### Expression of γ-glutamylcysteine synthetase (γ-GCS) and multidrug resistance-associated protein (MRP), but not human canalicular multispecific organic anion transporter (cMOAT), genes correlates with exposure of human lung cancers to platinum drugs

**T Oguri[1], Y Fujiwara[1,2], T Isobe[1], O Katoh[3], H Watanabe[3] and M Yamakido[1]**

[1]Second Department of Internal Medicine and [2]Department of Integrated Medicine, Hiroshima University School of Medicine; [3]Department of Environment and Mutation, Research Institute for Radiation Biology and Medicine, Hiroshima University, Kasumi 1-2-3, Minami-ku, Hiroshima 734, Japan

**Summary** We examined the steady-state levels of mRNA for γ-glutamylcysteine synthetase (γ-GCS), multidrug resistance-associated protein (MRP) and human canalicular multispecific organic anion transporter (cMOAT) in human lung cancer specimens to elucidate their roles in relation to platinum drug resistance in vivo. Seventy-six autopsy samples (38 primary tumours and their corresponding normal lung tissues) obtained from 38 patients were analysed using the quantitative reverse transcription polymerase chain reaction (RT-PCR) method. Both subunits (heavy and light subunits) of γ-GCS expression levels of normal lung and tumour tissues exposed to platinum drugs during life were significantly higher than those of non-exposed tissues, whereas only the MRP expression levels of tumours were elevated in association with ante-mortem platinum drug exposure. The γ-GCS and MRP expression levels correlated significantly. The cMOAT expression levels did not correlate with ante-mortem platinum drug exposure. Next, we monitored γ-GCS heavy subunit expression levels in peripheral mononuclear cells of eight previously untreated lung cancer patients after platinum drug administration, which revealed that these drugs induced γ-GCS expression in vivo. These results suggest that γ-GCS expression is induced by platinum drugs in vivo and/or the physiological stress response to xenobiotics.

**Keywords:** γ-GCS heavy subunit; γ-GCS light subunit; multidrug resistance-associated protein; canalicular multispecific organic anion transporter; drug resistance

Platinum drugs are used widely for chemotherapy of lung cancer, but their effectiveness is limited by the development and/or the de novo existence of resistance to them. Increased intracellular glutathione (GSH) levels and/or reduced drug accumulation have been reported to play important roles in platinum drug resistance (Bungo et al, 1990; Fujiwara et al, 1990; O'Brien and Tew, 1996).

The rate-limiting step enzyme for de novo GSH synthesis, i.e. γ-glutamylcysteine synthetase (γ-GCS), has been shown to be a crucial determinant of the platinum drug sensitivity of tumour cells (Bailey et al, 1992; Godwin et al, 1992; Mulcahy et al, 1994). Furthermore, several authors have confirmatively shown that γ-GCS overexpression caused in vitro drug resistance by virtue of increased GSH levels using the expression–vector transfection technique (Kurokawa et al, 1995; Mulcahy et al, 1995).

The precise mechanism responsible for membrane transport of platinum drugs is uncertain, but the ATP-dependent glutathione *S*-conjugate export (GS-X) pump has been shown to play an important role in their membrane transport and the acquisition of resistance to them (Ishikawa and Ali-Osman, 1993; Fujii et al, 1994; Ishikawa et al, 1994; Goto et al, 1995). Although the molecular structure of the GS-X pump has not been identified, several investigators have suggested that it is identical to the multidrug resistance-associated protein (MRP) (Jedlitschky et al, 1994; Leier et al, 1994; Müller et al, 1994) or that MRP is one of several members of a GS-X pump family (de Vries et al, 1995; Chuman et al, 1996). Furthermore, the recently cloned human canalicular multispecific organic anion transporter (cMOAT), a new ATP-binding cassette (ABC) transporter superfamily, is another candidate for the GS-X pump (Büchler et al, 1996; Paulusma et al, 1996; Taniguchi et al, 1996).

In these previous studies, however, only tumour cell lines were investigated extensively. Therefore, in this study, we examined the steady-state levels of mRNA for the γ-GCS, MRP and cMOAT in human lung cancer specimens to elucidate their roles in relation to platinum drug resistance in vivo.

### MATERIALS AND METHODS

### Patients and samples

Seventy-six autopsy samples (38 primary tumours and their corresponding normal lung tissues) from 38 patients with lung cancer admitted to Hiroshima University and Chugoku Rousai General Hospitals from June 1992 to October 1996 were studied. Fresh specimens of primary lung tumours and normal lung tissues were obtained during autopsy after informed consent had been obtained.

*Received 27 March 1997*
*Revised 10 July 1997*
*Accepted 3 September 1997*

*Correspondence to:* Y Fujiwara, Second Department of Internal Medicine, Hiroshima University School of Medicine, Kasumi 1-2-3, Minami-ku, Hiroshima 734, Japan

The tumour specimens were not contaminated by necrotic parts and normal lung tissues. The tissues were frozen in liquid nitrogen and stored at –80°C until analysed.

In order to examine gene induction in the human body after chemotherapy, peripheral mononuclear cells (PMN) were obtained from eight previously untreated lung cancer patients, who gave their informed consent. These patients were treated with cisplatin + irinotecan hydrochloride (CPT-11), cisplatin + etoposide or carboplatin + etoposide. They were all men and smokers, five of whom had small-cell lung cancer (SCLC) and three of whom had non-small-cell lung cancer (NSCLC); their ages ranged from 41 to 69 years (median 59 years). Five-millilitre heparinized blood samples were taken just before (0 h) and 6 and 24 h after completing platinum drug administration, and the PMN were separated immediately using lymphocyte preparation medium (Lymphoprep, Nycomed Pharma, Oslo, Norway), as described previously (Yao et al, 1993).

### RNA extraction and reverse transcriptase (RT) reaction

Total cellular RNA was extracted using the guanidinium isothiocyanate–phenol method described previously (Ohashi et al, 1996). We confirmed that the amount of total cellular RNA extracted from each sample showed almost the same quantity by ethidium bromide staining. cDNA was synthesized using random hexamer (Amersham, Buckinghamshire, UK) with Superscript RNase H- reverse transcriptase (Gibco, Bethesda, MD, USA), as described previously (Ohashi et al, 1996).

### Polymerase chain reaction (PCR) amplification

First, to confirm the harvested RNA quality, the reverse-transcribed cDNA synthesized from the same amount of total RNA in each sample was subjected to PCR amplification using β-actin primers. The sequences of its primers were: forward 5′-AAGAGAGGCATCCTCACCCT-3′ and reverse 5′-TACATGGCTGGGGTGTTGAA-3′. The PCR conditions were as described previously (Ohashi et al, 1996). Twenty amplification cycles using these primers were carried out, and the PCR products were 218 bp long, corresponding to β-actin cDNA. We found that expression levels of β-actin from each sample were about the same degree by ethidium bromide staining, even if it had been obtained from patients whose intervals between death and autopsy differed. Therefore, we considered that the quality of harvested RNA from our samples was acceptable for molecular analysis.

The reverse-transcribed cDNA from each sample was subjected to PCR amplification using primers based on the γ-GCS heavy subunit (γ-GCSh), γ-GCS light subunit (γ-GCSl), MRP, cMOAT and β-actin (internal control) gene sequences. After pre-denaturation at 94°C for 5 min, the cDNA was added to 5 μl of PCR mixture, comprising 1 μl of 10 × PCR buffer (100 mM Tris-HCl, pH 9.0, 500 mM potassium chloride), 1 μl of 15 mM magnesium chloride, 2 μl of distilled water, 0.2 μl of 20 mM dNTPs (Takara, Tokyo, Japan), 0.2 μl of 50 pM forward primer, 0.2 μl of 50 pM reverse primer and 0.4 μl (0.2 u) of *Taq* polymerase (Promega, Madison, WI, USA). The full-length coding regions of the γ-GCSh and γ-GCSl cDNAs of human liver γ-GCS have been sequenced (Gipp et al, 1992, 1995), and we designed and synthesized PCR primers for both subunits of human liver γ-GCS cDNA with the assistance of Advanced Gene Computing Technologies (Irvine, CA, USA; Mitsuhashi et al, 1994). The sequences of the used primers were as follows: γ-GCSh forward 5′-AGGCCAGATACCTTTATGATCAGT-3′ and reverse 5′-GCTGTCTATTGAGTCATATCGGGATTTAC-3′; γ-GCSl forward 5′-TGTCTTGGAATGCACTGTATCTCATGC-3′ and reverse 5′-TTCAATAGGAGGTGAAGCAATGATCAC-3′. We used the MRP primer as described previously (Ishikawa et al, 1996) and designed and synthesized the cMOAT primer ourselves. The sequences of the MRP and cMOAT primers were: MRP forward 5′-TGGGACTGGAATGTCACG-3′ and reverse 5′-AGGAATATGCCCCGACTTC-3′; cMOAT forward 5′-CTAATCTAGCCTACTCCTGC-3′ and reverse 5′-CTGCAGCTCTCTCTTCATGTGC-3′. All the PCR products were ligated with plasmid vectors and amplified using competent cells. The sequences of their nucleotides were identical to the corresponding partial cDNA sequences.

Amplification was carried out using a thermal cycler (Geneamp PCR System 2400; Perkin Elmer Applied Biosystems Division, Norwalk, CO, USA). Each amplification cycle for the reactions using the γ-GCSh, γ-GCSl and cMOAT primers comprised denaturation at 94°C for 30 s, annealing at 64°C for 30 s and extension at 72°C for 1 min, followed by a final incubation at 72°C for 7 min, whereas each cycle for amplification with the MRP primer comprised denaturation at 94°C for 30 s, annealing at 60°C for 30 s and extension at 72°C for 1 min, followed by a final incubation at 72°C for 7 min. The PCR products were 221, 196, 293 and 275 base pairs (bp) long, corresponding to γ-GCSh, γ-GCSl, MRP and cMOAT cDNA respectively.

In order to determine the optimal number of cycles, the accuracy of the quantitative PCR procedure was tested in titration experiments, as described previously (Ohashi et al, 1996). The optimal number of cycles for γ-GCSh and γ-GCSl was 22 and for MRP and cMOAT was 24. We used the β-actin gene as the internal control, and the sequences of its primers and amplification cycles were as described above.

### Quantitation of PCR products and analysis of mRNA expression

The PCR products were electrophoresed using 2% (w/v) agarose gels, transferred to nylon membranes (Hybond $N^+$; Amersham) and subjected to hybridization analysis with $^{32}P$-labelled cDNA probes using procedures described previously (Ohashi et al, 1996). After washing each filter, the radioactivity level was measured with a laser imaging analyser (BAS-2000; Fuji Photo Film, Tokyo, Japan). We used the full-length coding region of human γ-GCSh cDNA as the γ-GCSh probe (Kurokawa et al, 1995). The PCR products of MRP and cMOAT described above were used as the cDNA probes, and we designed and synthesized PCR primers for γ-GCSl and β-actin probes with the following sequences: γ-GCSl forward primer, as above, and reverse primer 5′-AAGTTTTCTAATTCCTCCCAGTAAGGC-3′; β-actin forward primer, as above, and reverse primer 5′-AATGGTGATGACCTGGCCGT-3′. The PCR products were 255 and 580 bp long, corresponding to γ-GCSl and β-actin cDNA, respectively, and they were both subcloned and sequenced as described above.

The radioactivity associated with expression levels in each sample was expressed relative to that due to β-actin expression levels in that sample.

### Statistical analysis

Contingency table analyses based on $\chi^2$ statistics were used to determine the significance of associations between categorical

*© Cancer Research Campaign 1998*

**Table 1** Patient characteristics

| Patient no. | Sex[a] | Age (years) | Smoke[b] | Type[c] | Therapy[d] | Pt dose (mg)[e] | Last Pt to death (months)[f] | Death to autopsy (h)[g] |
|---|---|---|---|---|---|---|---|---|
| 1 | M | 73 | + | SQ | | | | 11 |
| 2 | F | 51 | – | SM | CDDP+VP, CAV, RT 49.6 Gy | 169 | 8 | 2 |
| 3 | M | 68 | + | SQ | CBDCA | 3225 | 4 | 2 |
| 4 | M | 61 | + | SQ | CBDCA+VDS | 236 | 10 | 2 |
| 5 | M | 68 | + | SM | CBDCA+VP, CDDP+VP, CPT-11, CBDCA, RT 18 Gy | 790 | 4 | 1.5 |
| 6 | M | 50 | + | AD | CBDCA+VP | 315 | 1 | 2 |
| 7 | M | 51 | + | AD | CBDCA+VP, CBDCA | 2520 | 2 | 3 |
| 8 | M | 62 | + | AD | | | | 2.5 |
| 9 | M | 64 | + | AD | CBDCA+VP, RT 60 Gy | 227 | 10 | 15 |
| 10 | M | 64 | + | SM | CDDP+VP, CBDCA, RT 39.6 Gy | 1472 | 2 | 6 |
| 11 | M | 59 | + | SQ | CDDP, CBDCA+VP | 1157 | 2 | 14 |
| 12 | M | 61 | + | AD | CBDCA, RT 44 Gy | 1470 | 10 | 2 |
| 13 | F | 61 | – | AD | CBDCA, CDDP, RT 48.6 Gy | 525 | 5 | 1 |
| 14 | M | 74 | + | SM | CDDP+VP | 39 | 1 | 13 |
| 15 | M | 63 | + | SM | CDDP+CPT-11 | 270 | 5 | 13 |
| 16 | M | 67 | + | LA | RT 12 Gy | | | 7 |
| 17 | F | 44 | – | AD | CBDCA+VP, Topotecan, RT 60 Gy | 578 | 15 | 15 |
| 18 | M | 70 | + | SM | CDDP+VP | 267 | 5 | 2 |
| 19 | M | 68 | + | SQ | CDDP+VDS | 78 | 4 | 16 |
| 20 | F | 73 | + | AD | | | | 12 |
| 21 | F | 69 | + | SM | CDDP+CPT-11, CAE | 60 | 11 | 1.5 |
| 22 | M | 69 | + | AD | | | | 15 |
| 23 | M | 69 | + | AD | CDDP+VDS, CBDCA, RT 50 Gy | 393 | 24 | 13.5 |
| 24 | M | 57 | + | AD | CDDP+VDS | 85 | 4 | 2 |
| 25 | M | 60 | + | AD | Taxol, CDDP+CPT-11 | 114 | 7 | 4 |
| 26 | M | 71 | + | SM | | | | 6 |
| 27 | M | 57 | + | SQ | CDDP+VDS, CDDP+CPT-11, RT 50 Gy | 269 | 1 | 1 |
| 28 | F | 77 | + | AD | RT 9 Gy | | | 1 |
| 29 | F | 77 | + | SM | VP | | | 2 |
| 30 | M | 65 | + | AD | Taxol, CDDP+CPT-11, CDDP+5-FU, VP, RT 21 Gy | 137 | 7 | 3 |
| 31 | M | 86 | + | AD | | | | 2 |
| 32 | M | 69 | + | SM | CBDCA+VP | 326 | 1 | 9 |
| 33 | M | 78 | + | SQ | CBDCA+VP, RT 50 Gy | 630 | 3 | 2 |
| 34 | F | 82 | + | SM | CBDCA+VP, VP, RT 45 Gy | 315 | 12 | 12 |
| 35 | M | 69 | + | SQ | | | | 2 |
| 36 | M | 70 | + | AD | CDDP+VDS, RT 45 Gy | 195 | 36 | 4 |
| 37 | M | 82 | + | AD | | | | 2 |
| 38 | M | 65 | + | AD | | | | 2 |

[a]M, male; F, female. [b]+, patient who had smoked; –, patient who had never smoked. [c]SM, small-cell carcinoma; AD, adenocarcinoma; SQ, squamous cell carcinoma; LA, large-cell carcinoma, [d]CDDP, cisplatin; CBDCA, carboplatin; VP, etoposide; VDS, vindesine; CPT-11, irinotecan hydrochloride; Taxol, paclitaxel; 5-FU, 5-fluorouracil; CAV, cyclophosphamide + doxorubicin + vincristine; CAE, cyclophosphamide + doxorubicin + etoposide; RT, irradiation. [e]Total platinum dose. [f]Interval between final platinum drug administration and death (months). [g]Interval between death and autopsy (hours).

**Table 2** The steady stated mRNA levels of four genes

| Gene | Platinum exposed[a] | Platinum not exposed[b] | *P*-value |
|---|---|---|---|
| γ-GCSh | | | |
| Normal lung | 0.184 (0.004–4.525) | 0.024 (0.003–0.147) | 0.0017 |
| Tumour | 0.253 (0.005–6.106) | 0.021 (0.004–0.400) | 0.0008 |
| γ-GCSl | | | |
| Normal lung | 0.061 (0.006–3.195) | 0.032 (0.021–0.060) | 0.0080 |
| Tumour | 0.077 (0.012–1.541) | 0.028 (0.020–0.071) | 0.0001 |
| MRP | | | |
| Normal lung | 0.274 (0.023–2.777) | 0.177 (0.063–0.428) | NS[c] |
| Tumour | 0.306 (0.055–3.065) | 0.160 (0.016–0.821) | 0.0291 |
| cMOAT | | | |
| Normal lung | 0.233 (0.072–0.937) | 0.148 (0.075–0.310) | NS |
| Tumour | 0.205 (0.053–0.570) | 0.147 (0.048–0.309) | NS |

The steady state mRNA levels of four genes. All data are expressed as medians and ranges, and *P*-values < 0.05 were considerd to be significant. [a]Group of ante-mortem platinum drug exposed samples. [b]Group of ante-mortem platinum drug non-exposed samples. [c]Not significant.

© Cancer Research Campaign 1998

variables. Differences between the expression levels of each gene in tissue samples exposed and not exposed to platinum drugs were analysed using the Mann–Whitney *U*-test. All the gene expression levels were skewed toward higher expression levels and were subjected to logarithmic transformation so that they approximated more closely to a normal distribution, and then parametric tests (repeated measures of ANOVA, Pearson's correlation analysis) were performed. The statistical calculations and tests were performed using Stat View J4.11 Software (Abacus, CA, USA) and a Macintosh computer. All the statistical tests were two-sided, the data were expressed as medians and ranges and differences at *P*-values of less than 0.05 were considered to be significant.

**Figure 1** Steady-state mRNA levels for each gene are expressed relative to those of β-actin expression using the RT-PCR method, and the relationships of (**A**) γ-GCSh, (**B**) γ-GCSl, (**C**) MRP, (**D**) cMOAT and (**E**) Topo I expression levels between paired individual tumour and normal lung samples are shown. Open and solid circles denote tissues exposed and not exposed to platinum drugs ante-mortem respectively

© Cancer Research Campaign 1998

### RESULTS

### Patient characteristics

We analysed 76 autopsy samples (38 primary tumours and their corresponding normal lung tissues) from 38 patients using the RT-PCR method. The patient characteristics are presented in Table 1. There were 30 men and eight women; 11 had SCLC, 27 had NSCLC and they ranged in age from 44 to 86 years old (median 68 years). Almost all of them (35 of 38) had been smokers and 26 had received platinum drug therapy for their tumours.

### Relationships between platinum drugs and patient characteristics

γ-GCSh, γ-GCSl, MRP and cMOAT gene transcripts were detected in all the samples tested. In order to investigate the associations between previous platinum drug exposure and expression of each of these genes, we grouped the results according to whether the patient had or had not been exposed to these agents ante mortem. There were no significant differences in ages, sex, smoking histories, histology, platinum doses received, intervals between the last platinum drug administration and death, or intervals from death to autopsy between each group (data not shown).

### The steady-state levels of mRNA for both subunits of γ-GCS

We examined the relationships between previous platinum drug exposure and the steady-state levels of mRNA for both heavy and light subunits of γ-GCS. As shown in Table 2, the γ-GCSh expression levels were higher in exposed samples than in non-exposed samples (normal lung, $P = 0.0017$; lung tumour, $P = 0.0008$), and the γ-GCSl expression levels were also higher in exposed samples than in non-exposed (normal lung, $P = 0.0080$; lung tumour, $P = 0.0001$). There were significant correlations between the expression levels of both γ-GCS subunits in normal lung and lung tumour samples (γ-GCSh: $r = 0.847$, $P < 0.0001$; γ-GCSl: $r = 0.817$, $P < 0.0001$; Figure 1). Furthermore, γ-GCSh expression correlated with γ-GCSl expression in both normal lung ($r = 0.779$, $P < 0.0001$) and lung tumour ($r = 0.691$, $P < 0.0001$) samples. There were no significant correlations between the expression levels of either of both γ-GCS subunits and the cumulative platinum dose or the interval between the last platinum drug administration and death, and there were no significant differences between the expression levels of normal lung and tumour samples or between those of SCLC and NSCLC samples.

### The steady-state levels of mRNA for MRP and cMOAT

We also examined the relationships between previous platinum drug exposure and the steady-state levels of mRNA for MRP and cMOAT. As shown in Table 2, the MRP expression levels of the exposed group were higher than those of the non-exposed group only for the tumour samples ($P = 0.0291$). In contrast, there were no significant differences in cMOAT expression levels between the exposed and non-exposed groups of normal lung or tumour samples (Table 2). The expression levels of both MRP and cMOAT in normal and tumour tissue samples showed moderate correlations (MRP $r = 0.652$, $P = 0.0003$; cMOAT $r = 0.497$, $P = 0.0105$; Figure 1). Although, MRP expression correlated significantly with γ-GCSh expression in both normal lung ($r = 0.642$, $P = 0.0001$) and lung tumour ($r = 0.616$, $P = 0.0008$), the cMOAT expression showed no correlation with γ-GCSh expression. There were no significant correlations between the expression levels of either the MRP or the cMOAT and the cumulative platinum dose or interval between the last platinum drug administration and death. There were no differences between the expression levels of normal lung and those of tumour samples or between those of SCLC and NSCLC samples.

### The steady-state mRNA levels in normal liver

Next, we examined the relationships between previous platinum drug exposure and γ-GCSh, γ-GCSl, MRP and cMOAT in normal liver tissues of 24 patients, from whom we were able to obtain samples at the same time as the sampling of lung tissues. There were 16 exposed samples and eight non-exposed samples, and the median and range expression levels of liver samples were as follows: γ-GCSh, exposed 0.087 (0.004–0.396) and non-exposed 0.033 (0.002–0.073); γ-GCSl, exposed 0.134 (0.008–0.647) and non-exposed 0.081 (0.009–0.212); MRP, exposed 0.081 (0.024–0.399) and non-exposed 0.082 (0.038–0.153); cMOAT, exposed 0.203 (0.025–0.544) and non-exposed 0.180 (0.070–0.384). In contrast to the lung sample results, there were no differences between the platinum-exposed and -non-exposed groups in normal liver tissues (γ-GCSh, $P = 0.0982$; γ-GCSl, $P = 0.5815$; MRP, $P = 0.3583$; cMOAT, $P > 0.9999$).

### The steady-state levels of DNA topoisomerase I mRNA

As we considered that the mechanisms responsible for resistance to DNA topoisomerase I (Topo I) inhibitors and platinum drugs differ, we reanalysed the Topo I expression data (part of which was reported by Ohashi et al, 1996), according to the platinum drug exposure history. As shown in Figure 1E, there was no significant difference between the Topo I expression levels of the platinum-exposed and -non-exposed groups. The expression levels of Topo I in normal and tumour tissue samples showed significant correlation ($r = 0.928$, $P < 0.0001$; Figure 1E), although the Topo I expression showed no correlation with γ-GCSh expression.

**Table 3** The expression levels of γ-GCSh in peripheral mononuclear cells after platinum drug administration

| | Time after the completion of platinum drug infusion (h) | | |
|---|---|---|---|
| | 0 | 6 | 24 |
| γ-GCSh expression | 0.109 (0.079–0.176)[a] | 0.225 (0.105–0.752)[b] | 0.267 (0.176–0.533)[c] |

We monitored the γ-GCSH expression levels of PMN from eight patients with advanced lung cancer. The γ-GCSh expression levels increased after exposure ($P = 0.0048$, using repeated measures of ANOVA). [a]Each value is the median (range) of eight samples. [b]Median (range) of six samples (two samples were not available). [c]Median (range) of seven samples (one sample was not available).

© Cancer Research Campaign 1998 

### Induction of γ-GCSh expression in PMN

In order to determine whether platinum drugs actually induce γ-GCS expression levels in vivo, we monitored the γ-GCSh expression levels of PMN from eight patients with advanced lung cancer. As shown in Table 3, the γ-GCSh expression levels increased after exposure to platinum drug in a time-dependent manner ($P = 0.0048$, using repeated measures of ANOVA).

### DISCUSSION

This is, we believe, the first study to provide detailed data about the steady-state levels of mRNA for both subunits of γ-GCS, in conjunction with the steady-state levels of mRNA for MRP and cMOAT in clinical specimens. Our data demonstrated that the steady-state levels of mRNA for both subunits of γ-GCS and MRP, but not cMOAT, were significantly higher in tissues that had been exposed to platinum drugs in vivo than in those that had not.

In previous studies, the steady-state levels of γ-GCS mRNA in relation to drug resistance were investigated using cell lines in vitro (Bailey et al, 1992; Godwin et al, 1992; Mulcahy et al, 1994, 1995; Goto et al, 1995), and the results led to the general conclusion that increased expression resulted in drug resistance. The primary mechanism underlying this γ-GCS up-regulation-induced drug resistance was thought to be mediated through increased GSH levels. Furthermore, a recent transfection study showed that γ-GCS overexpression accompanied enhanced GS-X pump activity without increasing MRP expression (Kurokawa et al, 1995). Taken together, these and our results suggest that increased γ-GCS expression may be an index of both increased GSH levels and GS-X pump activity and therefore could be a useful drug resistance marker in a clinical setting.

To our knowledge, no detailed description of steady-state γ-GCSl mRNA levels in clinical samples has been reported, and therefore we could not compare our results with those of others. However, it is not surprising that γ-GCSl expression showed a highly significant correlation with γ-GCSh expression and ante-mortem platinum drug exposure history, because in vitro studies have demonstrated functional cooperation of γ-GCSh and γ-GCSl in GSH synthesis (Huang et al, 1993) and possibly in the GSH-mediated drug resistance mechanism (Mulcahy et al, 1995). Although γ-GCSl has tended to be overlooked by most investigators, our results demonstrate that it is important to investigate the expression levels of γ-GCSl as well as those of γ-GCSh.

In contrast to our results, Nooter et al (1996) have recently demonstrated that the steady-state MRP mRNA levels in NSCLC were higher than those in normal lung tissues. The reason for this discrepancy is uncertain because Nooter et al (1996) did not make it clear whether the normal lung tissues that they examined were obtained from the same patients as those whose lung cancer tissues were analysed.

Our results confirm those of Kuo et al (1996), who showed that γ-GCS and MRP expression was coordinated, possibly through a common transcriptional factor. In fact, *cis*-regulating elements including AP-1 binding sites are found to be present in the promoter regions of both genes (Zhu and Center, 1994; Mulcahy and Gipp, 1995; Yao et al, 1995), although whether both genes are regulated by AP-1 remains to be elucidated. As similar coordinated expression of γ-GCS and DT-diaphorase has been reported (O'Dwyer et al, 1996), these findings suggest the existence of an intricate regulatory network of xenobiotic detoxifying genes. Therefore, the changes in the level of MRP expression could synergize with the increases in the genes encoding γ-GCS and might result in platinum drug resistance. However, recent in vitro findings from other laboratories have suggested that overexpression of MRP does not induce cisplatin resistance (Grant et al, 1994) and that MRP is one of several members of the GS-X pump, but not the GSH–platinum conjugate export pump (Kurokawa et al, 1995; Chuman et al, 1996). These data indicate that further studies are required to determine the relationship between MRP and GSH, including the role of γ-GCS, in platinum drug resistance.

We detected no clear dose–response relationship between total platinum drug dose and the steady-state levels of γ-GCS and MRP mRNA. This may be because the threshold dose of such drugs required for gene induction is considerably lower than that usually administered clinically. Furthermore, it is noteworthy that the expression of each gene in normal liver tissues that had and had not been exposed to platinum drug ante mortem were virtually identical. These results suggest that transcriptional regulation of these xenobiotic detoxifying genes is tissue specific.

The cMOAT gene is a newly discovered member of the ABC transporter superfamily (Taniguchi et al, 1996) and has been suggested to participate in platinum drug transport. However, we observed no association between ante-mortem platinum drug exposure and the steady-state cMOAT mRNA level. Although the precise role of cMOAT in platinum drug sensitivity needs further investigation, our results suggest it does not play a major role in platinum drug transport and/or resistance.

One criticism of our study is that we used autopsy samples. However, all the autopsies were performed within 16 h of death, which has been proved to be acceptable for obtaining mRNAs and proteins of satisfactory quality (Kleiner et al, 1995). As human tissue is not only a valuable but also a limited resource for research outside surgery and pathology departments, we think that molecular analysis of autopsy samples should be encouraged.

Over half (16 of 27) of our patients who received platinum drug therapy were also given etoposide as a part of a combination regimen. As etoposide has been found to persist in tissues for a few days (Stewart et al, 1993), and our autopsy samples were taken several months after the final anti-cancer drug administration, we think that it is difficult to evaluate the impact of etoposide co-administration on the expression of the four genes that we examined.

Furthermore, we detected no differences among the steady-state levels of γ-GCS, MRP or cMOAT mRNA in tumour and normal lung tissues, suggesting that their increased expression after exposure to platinum drugs is part of the normal stress response to xenobiotics. Kondo et al (1993) reported that treatment of K-562 cells in culture with heavy metals induced a high level of γ-GCSh expression and that the response to this stress was similar to that after heat shock stress. Furthermore, Ishikawa et al (1996) reported that cisplatin induced γ-GCSh expression in cisplatin-resistant human leukaemia HL-60 cells. In accordance with these in vitro results, we observed that γ-GCSh expression level in PMN increased rapidly after exposure to platinum drugs in vivo. Whether the platinum drug or platinum itself is responsible for this in vivo gene induction remains to be elucidated. Although residual platinum may induce γ-GCSh overexpression continuously, as platinum persists in tissues for several months to years after final exposure (Tothill et al, 1992), further studies are required to determine whether acutely exposed and chronically exposed tissues share a common regulatory mechanism of gene induction.

© Cancer Research Campaign 1998

### ABBREVIATIONS

GSH, glutathione; γ-GCS, γ-glutamylcysteine synthetase; γ-GCSh, γ-GCS heavy subunit; γ-GCSl, γ-GCS light subunit; GS-X pump, ATP-dependent glutathione *S*-conjugate export pump; MRP, multidrug resistance-associated protein; cMOAT, human canalicular multispecific organic anion transporter; Topo I, DNA topoisomerase I; PMN, peripheral mononuclear cells; RT-PCR, reverse transcription polymerase chain reaction; ABC, ATP-binding cassette

### ACKNOWLEDGEMENTS

This study was supported by Grants-in-Aid from the Ministry of Education, Science and Culture and the Ministry of Health and Welfare of Japan, the Japan Society for the Promotion of Science Fellowship for the Study at the Center of Excellence Abroad and a grant from Osaka Cancer Research Foundation and Chugai Seiyaku. We are grateful to Drs N Saijo and K Nishio (Pharmacology Division, National Cancer Center Research Institute) for providing the coding region of cDNA for the heavy subunit of human liver γ-GCS, Drs T Tsuya and T Ohune (Department of Respiratory Disease, Chugoku Rousai General Hospital) for providing autopsy samples, Dr N Ohashi (Department of Respiratory Disease, Hiroshima Red Cross Hospital and Atomic Bomb Survivors Hospital) for his practical suggestions during the study.

### REFERENCES

Bailey HH, Gipp JJ, Ripple M, Wilding G and Mulcahy RT (1992) Increase in γ-glutamylcysteine synthetase activity and steady-state messenger RNA levels in melphalan-resistant DU-145 human prostate carcinoma cells expressing elevated glutathione levels. *Cancer Res* **52**: 5115–5118

Büchler M, König J, Brom M, Kartenbeck J, Spring H, Horie T and Keppler D (1996) cDNA cloning of the hepatocyte canalicular isoform of the multidrug resistance protein, cMrp, reveals a novel conjugate export pump deficient in hyperbilirubinemic mutant rats. *J Biol Chem* **271**: 15091–15098

Bungo M, Fujiwara Y, Kasahara K, Nakagawa K, Ohe Y, Sasaki Y, Irino S and Saijo N (1990) Decreased accumulation as a mechanism of resistance to *cis*-diamminedichloroplatinum (II) in human non-small cell lung cancer cell lines: relation to DNA damage and repair. *Cancer Res* **50**: 2549–2553

Chuman Y, Chen Z-S, Sumizawa T, Furukawa T, Haraguchi M, Takebayashi Y, Niwa K, Yamada K, Aikou T and Akiyama S (1996) Characterization of the ATP-dependent LTC4 transporter in cisplatin-resistant human KB cells. *Biochem Biophys Res Commun* **226**: 158–165

De Vries EGE, Müller M, Meijer C, Jansen PLM and Mulder NH (1995) Role of the glutathione *S*-conjugate pump in cisplatin resistance. *J Natl Cancer Inst* **87**: 537–538

Fujii R, Mutoh M, Sumizawa T, Chen Z-S, Yoshimura A and Akiyama S (1994) Adenosine triphosphate-dependent transport of leukotriene C4 by membrane vesicles prepared from cisplatin-resistant human epidermoid carcinoma tumor cells. *J Natl Cancer Inst* **86**: 1781–1784

Fujiwara Y, Sugimoto Y, Kasahara K, Bungo M, Yamakido M, Tew KD and Saijo N (1990) Determinants of drug response in a cisplatin-resistant human lung cancer cell line. *Jpn J Cancer Res* **81**: 527–535

Gipp JJ, Chang C and Mulcahy RT (1992) Cloning and nucleotide sequence of a full-length cDNA for human liver γ-glutamylcysteine synthetase. *Biochem Biophys Res Commun* **185**: 29–35

Gipp JJ, Bailey HH and Mulcahy RT (1995) Cloning and sequencing of the cDNA for the light subunit of human liver γ-glutamylcysteine synthetase and relative mRNA levels for heavy and light subunits in human normal tissues. *Biochem Biophys Res Commun* **206**: 584–589

Godwin AK, Meister A, O'Dwyer PJ, Huang CS, Hamilton TC and Anderson ME (1992) High resistance to cisplatin in human ovarian cancer cell lines is associated with marked increase of glutathione synthesis. *Proc Natl Acad Sci USA* **89**: 3070–3074

Goto S, Yoshida K, Morikawa T, Urata Y, Suzuki K and Kondo T (1995) Augmentation of transport for cisplatin-glutathione adduct in cisplatin-resistant cancer cells. *Cancer Res* **55**: 4297–4301

Grant CE, Valdimarsson G, Hipfner DR, Almquist KC, Cole SPC and Deeley RG (1994) Overexpression of multidrug resistance-associated protein (MRP) increases resistance to natural product drugs. *Cancer Res* **54**: 357–361

Huang C-S, Chang L-S, Anderson ME and Meister A (1993) Catalytic and regulatory properties of the heavy subunit of rat kidney γ-glutamylcysteine synthetase. *J Biol Chem* **268**: 19675–19680

Ishikawa T and Ali-Osman F (1993) Glutathione-associated *cis*-diamminedichloroplatinum(II) metabolism and ATP-dependent efflux from leukemia cells. *J Biol Chem* **268**: 20116–20125

Ishikawa T, Wright CD and Ishizuka H (1994) *GS-X* pump is functionally overexpressed in *cis*-diamminedichloroplatinum (II)-resistant human leukemia HL-60 cells and down-regulated by cell differentiation. *J Biol Chem* **269**: 29085–29093.

Ishikawa T, Bao J-J, Yamane Y, Akimaru K, Frindrich K, Wright CD and Kuo MT (1996) Coordinated induction of *MRP/GS-X* pump and γ-glutamylcysteine synthetase by heavy metals in human leukemia cells. *J Biol Chem* **271**: 14981–14988

Jedlitschky G, Leier I, Buchholz U, Center M and Keppler D (1994) ATP-dependent transport of glutathione *S*-conjugates by the multidrug resistance-associated protein. *Cancer Res* **54**: 4833–4836

Kleiner DE, Emmert-Buck MR and Liotta LA (1995) Necropsy as a research method in the age of molecular pathology. *Lancet* **346**: 945–948.

Kondo T, Yoshida K, Urata Y, Goto S, Gasa S and Taniguchi N (1993) γ-glutamylcysteine synthetase and active transport of glutathione *S*-conjugate are responsive to heat shock in K562 erythroid cells. *J Biol Chem* **268**: 20366–20372

Kuo MT, Bao J-J, Curley SA, Ikeguchi M, Johnston DA and Ishikawa T (1996) Frequent coordinated overexpression of the *MRP/GS-X* pump and γ-glutamylcysteine synthetase genes in human colorectal cancers. *Cancer Res* **56**: 3642–3644

Kurokawa H, Ishida T, Nishio K, Arioka H, Sata M, Fukumoto H, Miura M and Saijo N (1995) γ-glutamylcysteine synthetase gene overexpression results in increased activity of the ATP-dependent glutathione *S*-conjugate export pump and cisplatin resistance. *Biochem Biophys Res Commun* **216**: 258–264

Leier I, Jedlitschky G, Buchholz U, Cole SPC, Deeley RG and Keppler D (1994) The MRP gene encodes an ATP-dependent export pump for leukotriene C4 and structurally related conjugates. *J Biol Chem* **269**: 27807–27810

Mitsuhashi M, Cooper A, Ogura M, Shinagawa T, Yano K and Hosokawa T (1994) Oligonucleotide probe design – a new approach. *Nature* **367**: 759–761

Mulcahy RT and Gipp JJ (1995) Identification of a putative antioxidant response element in the 5′-flanking region of the human γ-glutamylcysteine synthetase heavy subunit gene. *Biochem Biophys Res Commun* **209**: 227–233

Mulcahy RT, Bailey HH and Gipp JJ (1994) Up-regulation of γ-glutamylcysteine synthetase activity in melphalan-resistant human multiple myeloma cells expressing increased glutathione levels. *Cancer Chemother Pharmacol* **34**: 67–71

Mulcahy RT, Bailey HH and Gipp JJ (1995) Transfection of complementary DNAs for the heavy and light subunits of human γ-glutamylcysteine synthetase results in an elevation of intracellular glutathione and resistance to melphalan. *Cancer Res* **55**: 4771–4775

Müller M, Meijer C, Zaman GJR, Borst P, Scheper RJ, Mulder NH, De Vries EGE and Jansen PLM (1994) Overexpression of the gene encoding the multidrug resistance-associated protein results in increased ATP-dependent glutathione *S*-conjugate transport. *Proc Natl Acad Sci USA* **91**: 13033–13037

Nooter K, Bosman FT, Burger H, Van Wingerden KE, Flens MJ, Scheper RJ, Oostrum RG, Boersma AWM, Van Der Gaast A and Stoter G (1996) Expression of the multidrug resistance-associated protein (MRP) gene in primary non-small-cell lung cancer. *Ann Oncol* **7**: 75–81

O'Brien ML and Tew KD (1996) Glutathione and related enzymes in multidrug resistance. *Eur J Cancer* **32A**: 967–978

O'Dwyer PJ, Szarka CE, Yao K-S, Halbherr TC, Pfeiffer GR, Green F, Gallo JM, Brennan J, Frucht H, Goosenberg EB, Hamilton TC, Litwin S, Balshem AM, Engstrom PF and Clapper ML (1996) Modulation of gene expression in subjects at risk for colorectal cancer by the chemopreventive dithiolethione oltipraz. *J Clin Invest* **98**: 1210–1217

Ohashi N, Fujiwara Y, Yamaoka N, Katoh O, Satow Y and Yamakido M (1996) No alteration in DNA topoisomerase I gene related to CPT-11 resistance in human lung cancer. *Jpn J Cancer Res* **87**: 1280–1287

Paulusma CC, Bosma PJ, Zaman GJR, Bakker CTM, Otter M, Scheffer GL, Scheper RJ, Borst P and Oude Elferink RPJ (1996) Congenital jaundice in rats with a

© Cancer Research Campaign 1998

mutation in a multidrug resistance-associated protein gene. *Science* **271**: 1126–1128

Stewart DJ, Grewaal D, Redmond MD, Mikhael NZ, Montpetit VAJ, Goel R and Green RM (1993) Human autopsy tissue distribution of the epipodophyllotoxins etoposide and teniposide. *Cancer Chemother Pharmacol* **32**: 368–372

Taniguchi K., Wada M, Kohno K, Nakamura T, Kawabe T, Kawakami M, Kagotani K, Okumura K, Akiyama S, and Kuwano M (1996) A human canalicular multispecific organic anion transporter (cMOAT) gene is overexpressed in cisplatin-resistant human cancer cell lines with decreased drug accumulation. *Cancer Res* **56**: 4124–4129

Tothill P, Klys HS, Matheson LM, McKay K and Smyth JF (1992) The long-term retention of platinum in human tissues following the administration of cisplatin or carboplatin for cancer chemotherapy. *Eur J Cancer* **28**: 1358–1361

Yao K-S, Godwin AK, Ozols RF, Hamilton TC and O'Dwyer PJ (1993) Variable baseline γ-glutamylcysteine synthetase messenger RNA expression in peripheral mononuclear cells of cancer patients, and its induction by buthionine sulfoximine treatment. *Cancer Res* **53**: 3662–3666

Yao K-S, Godwin AK, Johnson SW, Ozols RF, O'Dwyer PJ and Hamilton TC (1995) Evidence for altered regulation of γ-glutamylcysteine synthetase gene expression among cisplatin-sensitive and cisplatin-resistant human ovarian cancer cell lines. *Cancer Res* **55**: 4367–4374

Zhu Q and Center MS (1994) Cloning and sequence analysis of the promoter region of the MRP gene of HL60 cells isolated for resistance to adriamycin. *Cancer Res* **54**: 4488–4492

*© Cancer Research Campaign 1998*